# Special Issue “Molecular Advances in Dry Eye Syndrome”

**DOI:** 10.3390/ijms262411863

**Published:** 2025-12-09

**Authors:** Anxo Fernández-Ferreiro, Maria Jesus Giráldez-Fernández

**Affiliations:** 1 FarmaCHUSLab Group, Health Research Institute of Santiago de Compostela (IDIS), 15706 Santiago de Com-postela, Spain; 2 Pharmacy Department, University Clinical Hospital of Santiago de Compostela (SERGAS), 15706 Santiago de Compostela, Spain; 3 Applied Physics Department (Optometry Area), Facultade de Óptica e Optometría, Universidade de Santiago de Compostela, 15705 Santiago de Compostela, Spain; 4 Optometry Group, Instituto de Investigación Sanitaria Santiago de Compostela (IDIS), 15706 Santiago de Compostela, Spain

Dry eye disease (DED) is a multifactorial and highly prevalent disorder of the ocular surface, characterized by tear film instability, ocular discomfort, inflammation, and visual fluctuation. Affecting millions worldwide, DED is increasingly recognized as a condition that significantly compromises patients’ quality of life and, in advanced stages, can lead to permanent visual impairment. Underlying the clinical manifestations of DED is a complex interplay of molecular, immunological, hormonal, and neurosensory mechanisms that have become the focus of intense research. While current therapies can address certain symptoms, many patients continue to experience persistent ocular surface inflammation, epithelial damage, and neurosensory dysfunction. Therefore, new insights into the molecular basis of DED, the development of improved diagnostic tools, and the identification of innovative pharmacological and biological therapies remain essential. In this Editorial, we summarize the primary findings presented in the articles published in this Special Issue, which aimed to expand current knowledge of DED pathophysiology and introduce new therapeutic perspectives. Recent advances consolidate the TFOS DEWS II framework, which defines DED as a loss of tear film homeostasis driven by tear instability, hyperosmolarity, inflammation, and neurosensory abnormalities. These guidelines emphasize the integration of molecular biomarkers, imaging modalities, and patient-reported outcomes to refine diagnosis and guide therapy, concepts that serve as the foundation for interpreting the mechanistic contributions of this Special Issue [1].

One of the central aspects explored in this Special Issue is the composition and function of the tear film, especially its lipid components. García-Queiruga and colleagues conducted a comprehensive lipidomic analysis of meibum in subjects with evaporative DED. In this study, the authors examined alterations in key lipid species associated with meibomian gland dysfunction. Their findings revealed significant changes in lipid classes relevant to tear film stability, providing molecular insight into the mechanisms that contribute to the evaporative subtype of DED. The detailed lipidomic signatures described by the authors offer potential avenues for future biomarker development and targeted therapeutic interventions, further highlighting the crucial role played by meibomian gland physiology in maintaining tear film homeostasis. Investigations have demonstrated that specific lipid species, such as O-acyl-ω-hydroxy fatty acids (OAHFAs), exhibit liquid-expanded to solid-phase transitions that create highly evaporation-resistant films, while diesters lack this protective capability. The tear film lipid layer serves as the primary barrier against aqueous evaporation, maintains a smooth optical surface, and is essential for ocular surface hydration and tear distribution. Notably, up to 86% of patients with dry eye disease show evidence of meibomian gland dysfunction, and evaporative DED is far more prevalent than pure aqueous deficiency in general clinical cohorts [2,3].

The ocular surface is also influenced by extracellular vesicles naturally present in tear fluids. Oya et al. examined the potential functions of murine tear-derived extracellular vesicles and investigated their effects on corneal epithelial cells in vitro. The authors demonstrated that these vesicles may modulate epithelial cell physiology and participate in intercellular communication. Their findings suggest that vesicles contained in tear fluid might contribute to epithelial repair processes or serve as biomarkers of ocular surface health. Emerging evidence supports the role of tear-derived and cell-derived extracellular vesicles as regulators of inflammation and epithelial repair. Systematic reviews highlight their utility as minimally invasive biomarkers in dry eye and Sjögren-associated keratoconjunctivitis sicca, while preclinical studies suggest that stem cell-derived exosomes may promote nerve regeneration and reduce ocular surface inflammation [4].

A second thematic focus of this Special Issue concerns advances in pharmacological and biological treatments for DED. In an open-label, sequential prospective study, Puente-Iglesias et al. evaluated the clinical effectiveness, safety, and compliance of two compounded formulations of tacrolimus eye drops. Forty patients received sequential treatment with both formulations, and the authors examined ocular surface signs, symptoms, and tolerability. Their results demonstrated notable improvements in clinical parameters and inflammatory signs, with acceptable safety profiles and positive adherence. Calcineurin inhibitors remain key immunomodulatory agents for chronic ocular surface inflammation. Clinical studies indicate that cyclosporine and tacrolimus achieve disease control in 52% and 62% of patients, respectively. Tacrolimus is pharmacodynamically 10–100 times more potent than cyclosporine and exhibits a favorable safety profile. In dry eye disease, it suppresses pro-inflammatory cytokines including IL-2, IFN-γ, and TNF-α, as demonstrated in preclinical models of ocular inflammation [5].

Novel biological approaches were also explored. Lee and colleagues investigated the anti-inflammatory properties of exosomes derived from *Limosilactobacillus fermentum* in a model of benzalkonium chloride-induced inflammation in conjunctival cells. Their work showed that these bacterial exosomes significantly reduced inflammation markers and cellular stress responses. Diquafosol, a well-known P2Y2 receptor agonist, was evaluated in a systematic review and meta-analysis by Serrano-Robles et al. In a randomized double-masked phase III clinical trial involving 287 adults with dry eye disease, 3% diquafosol ophthalmic solution demonstrated non-inferiority to 0.1% sodium hyaluronate and achieved superior improvements in corneal fluorescein staining, tear break-up time, and rose Bengal conjunctival staining. Long-term observational studies support its safety and sustained efficacy in real-world settings [6].

Kaštelan et al. reviewed sex-based differences in lacrimal gland anatomy, function, hormonal regulation, and immune responsiveness. Sexual dimorphism significantly influences lacrimal gland structure and aging. Women exhibit more pronounced degenerative changes—including fibrosis and acinar atrophy—particularly after menopause. Furthermore, androgen deficiency has been implicated in meibomian and lacrimal gland dysfunction, underscoring a hormonal basis for the increased susceptibility to dry eye disease observed in women [7].

Saram and colleagues provided a comprehensive overview of the immunobiology of DED, including the roles played by innate and adaptive immune responses, epithelial damage, cytokine networks, and emerging therapeutic targets. Likewise, Soyfoo et al. focused on the diagnostic and therapeutic challenges of Sjögren disease.

Neurosensory mechanisms also play a pivotal role in DED severity and chronicity. Kahuam-López et al. reviewed the significance of nerve growth factor (NGF) in ocular surface physiology and pathology. NGF is essential for immune modulation, trophic support, epithelial healing, corneal sensitivity, and tear film regulation. Experimental and clinical studies demonstrate that NGF accelerates the regeneration of sub-basal and stromal nerves after LASIK surgery and improves corneal sensitivity and tear stability. These findings support the development of NGF-based therapies such as cenegermin, now approved for neurotrophic keratitis [8]. Environmental exposures such as PHMG-p and air pollutants also contribute to ocular surface disease.

Valencia-Sandonís and colleagues monitored molecular and clinical changes in chronic DED with ocular pain. Growing evidence indicates that a persistent nociceptive input from the ocular surface can induce central sensitization, with alterations in pain pathways and a reduced response to conventional therapies. Patients with neuropathic-like symptoms often require multimodal approaches including neuromodulators and targeted anti-inflammatory strategies [9].

Overall, the contributions to this Special Issue highlight several key insights into dry eye disease: that lipidomic disturbances underpin evaporative DED; that tacrolimus and diquafosol offer promising therapeutic benefits; that microbial- and tear-derived vesicles have regulatory functions in ocular surface biology; that sex differences, immunological mechanisms, and neurosensory factors significantly shape disease presentation; that environmental toxicants may exacerbate ocular surface dysfunction; and that chronic inflammation and pain mechanisms persist in many patients despite treatment. The combination of these findings deepens our understanding of DED from diverse perspectives and paves the way for the performance of future research. In the future, long-term studies of new pharmacological agents, biologically derived therapies, and personalized diagnostic tools should be pursued in larger patient cohorts. Integrating multi-omics approaches, advanced imaging technologies, and artificial intelligence will be essential for identifying reliable biomarkers, refining classification systems, and developing individualized treatment strategies. The advances presented in this Special Issue not only expand our molecular and clinical understanding of DED but also open up new avenues for research and innovation that aims to improve therapeutic outcomes and enhance patients’ quality of life.
